# Association between plasma gamma-glutamyltransferase fractions and metabolic syndrome among hypertensive patients

**DOI:** 10.1038/s41598-017-12356-w

**Published:** 2017-09-20

**Authors:** Maria Franzini, Ilenia Scataglini, Angelo Ricchiuti, Vanna Fierabracci, Aldo Paolicchi, Alfonso Pompella, Giulia Dell’Omo, Roberto Pedrinelli, Alessandro Corti

**Affiliations:** 1 Department of Translational Research and New Technologies in Medicine and Surgery, University of Pisa, Pisa, Italy; 2 Azienda Ospedaliero Universitaria Pisana, Gastroenterology Unit, Pisa, Italy; 3 Department of Surgery, Medical, Molecular, and Critical Area Pathology, University of Pisa, Pisa, Italy

## Abstract

Among the risk factors associated to metabolic syndrome (MetS), hypertension shows the highest prevalence in Italy. We investigated the relationship between the newly identified serum γ-glutamyltransferase (GGT) fractions, b- s- m- f-GGT, and risk factors associated to MetS in hypertensive patients. A total of ninety-five consecutive hypertensive patients were enrolled. GGT fractions were analysed by gel-filtration chromatography, and hepatic steatosis was evaluated by ultrasound. MetS was diagnosed in 36% of patients. Considering the whole group, b- and f-GGT showed the highest positive correlation with BMI, glucose, triglycerides and insulin, and the highest negative correlation with HDL cholesterol. While both serum triglycerides and insulin were independently associated with b-GGT levels, only triglycerides were independently associated with f-GGT. The values of b-GGT activity increased with steatosis grade (g0 = 1.19; g2 = 3.29; *ratio* g2/g0 = 2.75, p < 0.0001 linear trend). Patients with MetS showed higher levels of b-GGT, m-GGT and f-GGT [median (25^th^–75^th^) U/L: 3.19 (1.50–6.59); 0.55 (0.26–0.81); 10.3 (9.1–13.6); respectively] as compared to subjects presenting with one or two MetS criteria [1.75 (0.95–2.85), p < 0.001; 0.33 (0.19–0.60), p < 0.05; 8.8 (7.0–10.6), p < 0.001]. Our data point to a potential role for b- and f-GGT fractions in identifying MetS patients among hypertensive subjects, thus providing a minimally invasive blood-based tool for MetS diagnosis.

## Introduction

Metabolic syndrome (MetS) is a complex clinical condition represented by a cluster of five interconnected risk factors including abdominal obesity, insulin resistance, high levels of serum triglycerides, low high-density lipoprotein (HDL) cholesterol and hypertension. The presence of three or more of these factors allows the clinical diagnosis of MetS^1^. MetS is associated with a 5-fold increased risk of type 2 diabetes mellitus, 3-fold increased risk of cardiovascular disease and also with an increased risk of developing some types of cancers^2,3^. MetS is a growing problem worldwide and substantial efforts have been made in the last years to identify early, minimally invasive blood-based biomarkers for MetS diagnosis. Indeed, a large number of biomarkers have been reported to be associated – even not exclusively – with MetS^4^ and recent studies have aimed at the identification of early biomarkers of MetS – such as specific levels/types of extracellular microvesicles, DNA, RNAs or proteins^5^.

Serum γ-glutamyltransferase (GGT; EC 2.3.2.2) has been proposed as a useful predictive biomarker for MetS, as its levels were found to correlate with an increased risk of metabolic syndrome and type 2 diabetes^6^. The Framingham Heart Study demonstrated that GGT is positively associated with body mass index, blood pressure, LDL cholesterol, triglycerides, and blood glucose (*i.e*. all the major components of MetS) and that the risk of metabolic syndrome increases with higher GGT levels^7^. Other large population studies confirmed the association between GGT and MetS^8,9^ further supporting the potential role of GGT for a better classification of patients diagnosed with MetS.

Nevertheless, serum GGT activity is firstly recognized as a sensitive marker of liver dysfunction and alcohol abuse, though with a low specificity. GGT levels increase in various physiological and pathological conditions including hepatobiliary disorders such as steatosis and viral hepatitis^10^.

A high sensitivity clinical laboratory method allowing the simultaneous detection of four different fractions of GGT in human plasma has been developed in our laboratories^11^. These fractions consist of three GGT-containing molecular complexes, *i.e*. b-GGT, m-GGT, and s-GGT, with molecular weight >2000, 940, 140 kDa, respectively, and the free enzyme, f-GGT (70 kDa). We found that f-GGT is the most abundant fraction in healthy subjects^12^, while s-GGT increases to become the main GGT fraction in chronic viral hepatitis C and alcoholic-liver disease^13,14^ and that b-GGT levels are elevated in non-alcoholic fatty liver disease (NAFLD)^13^. The b-GGT fraction is also positively associated with several cardiovascular risk factor, including atherogenic dyslipidemia^15^. Patients from the Framingham Heart Study showed a positive correlation between markers of MetS (BMI, DBP, glucose, triglycerides) and the b- and f-GGT fractions, with an increase of the b/s ratio^15^.

As regards Italy, among the risk factors associated to MetS hypertension shows the highest prevalence^16,17^. Hypertension is known to be associated with insulin resistance and thus with alterations in glucose homeostasis which are the main features of MetS^18,19^.

The aim of the present investigation was to establish if a specific GGT fraction pattern is associated with MetS in a population of hypertensive patients at high risk for cardiovascular disease.

## Patients and Methods

### Patient selection

Ninety-five consecutive Caucasian patients presenting with hypertension and one or more additional risk factors for the diagnosis of MetS were enrolled in the study at the O.U. of Cardiovascular Disease (University Hospital of Pisa; Department of Surgery, Medical, Molecular, and Critical Area Pathology). MetS was defined using modified National Cholesterol Education Program (NCEP) criteria^1,7^, which requires at least three of the following: elevated blood pressure (≥130 mmHg systolic, ≥85 mmHg diastolic) or anti-hypertensive drug treatment; elevated fasting blood glucose (≥100 mg/dL) or drug treatment for elevated glucose; high triglyceride levels (≥150 mg/dL), reduced HDL cholesterol levels (<50 mg/dL for women and <40 mg/dL for men), high body mass index (BMI ≥ 27.2 for women and ≥29.5 for men). Grading of diffuse hepatic steatosis was evaluated by liver ultrasound and patients were grouped depending on their steatosis grade (0 = no steatosis; 1 = low; 2 = medium; 3 = high).

The following represented exclusion criteria: presence of liver disease (*i.e*. viral hepatitis, cholestasis, hepatocellular carcinoma, cirrhosis); excessive alcohol consumption (more than 45 g/day for men or 30 g/day for women, according to World Health Organization Recommendations 2014); use of hepatotoxic drugs.

The Institutional Ethics Committee of the University Hospital of Pisa approved the study (n° 3865; date: 12/11/2013) and all subjects gave informed consent. All methods were performed in accordance with the relevant guidelines and regulations.

### Liver ultrasound

Ultrasound (US) examinations were performed by using a US unit (ACUSON S2000TM Siemens), with a 3–4.5 MHz convex array transducer. Patients were examined following an overnight fasting period. Hepatic steatosis on US appears as a diffuse increase in hepatic echogenicity, or “bright liver”, due to increased reflection of US from the liver parenchyma, which is caused by intracellular accumulation of fat vacuoles. US evaluation of hepatic steatosis typically consists of a qualitative visual assessment of hepatic echogenicity, measurements of the difference between the liver and kidneys in echo amplitude, evaluation of echo penetration into the deep portion of the liver, and determination of the clarity of blood vessel structures in the liver^20^.

The alteration of echogenicity was graded as follows: grade 0, normal echogenicity; grade 1, slight, diffuse increase in fine echoes in liver parenchyma with normal visualization of diaphragm and intrahepatic vessel borders; grade 2, moderate, diffuse increase in fine echoes with slightly impaired visualization of intrahepatic vessels and diaphragm; grade 3, marked increase in fine echoes with poor or no visualization of the intrahepatic vessel borders, diaphragm, and posterior right lobe of the liver^21^.

### Laboratory analyses

Standard assay of all blood tests were simultaneously performed according to the standard clinical laboratory procedures by automated analyzers, and included: creatinine, glucose, insulin, total cholesterol; low density lipoprotein (LDL) cholesterol; high density lipoprotein (HDL) cholesterol, triglycerides (TG); total and direct bilirubin; aspartate aminotransferases (AST); alanine aminotransferases (ALT); alkaline phosphatases (ALP), C-reactive protein (CRP), complete blood count. Analysis were performed at the Clinical Laboratory of the University Hospital of Pisa; quality control was ensured by the participation to external quality assessment of the Tuscany Region (Italy).

### Gamma-glutamyltransferase fraction analysis

Analysis of total and fractional GGT was performed, as previously described^11,12^, on plasma-EDTA samples using a fast protein liquid chromatography system (AKTA purifier; GE Healthcare Europe, Milan, Italy) equipped with a gel-filtration column (Superose 6 HR 10/300 GL; GE Healthcare Europe) and a fluorescence detector (Jasco FP-2020; Jasco Europe, Lecco, Italy). Separation of fractional GGT was obtained by gel-filtration chromatography and the enzymatic activity was quantified by post-column injection of the fluorescent substrate for GGT, gamma-glutamyl-7-amido-4-methylcoumarin (gGluAMC). Enzymatic reaction, in the presence of gGluAMC 0.030 mmol/L and glycylglycine 4.5 mmol/L, proceeded for 4.5 min in a reaction coil (PFA, 2.6 mL) kept at the 37 °C in a water bath. The fluorescence detector operating at excitation/emission wavelengths of 380/440 nm detected the AMC signal; the intensity of the fluorescence signal was expressed in arbitrary fluorescence units. Under this reaction conditions, area under curve (AUC) is proportional to GGT activity. Fractional GGT activity was quantified as previously described^12^.

### Statistical analysis

Data are presented as mean (standard deviation, SD) or median (25^th^-75^th^ percentile) as appropriate. GGT fractions, the b-GGT/s-GGT ratio, glucose, TG, ALT and insulin data were ln-transformed to reduce the distribution skewness. The statistical comparisons between two or more groups were carried out with the Student’s t-test or one-way analysis of variance (ANOVA), followed by post test for linear trend, respectively. Two-way ANOVA was used to test the effect of MetS presence (categorized as presence/absence) and the grade of steatosis (categorized as grade 0, 1, 2, 3). Univariate linear correlations between variables and fractional GGT activity were evaluated with the Pearson’s correlation coefficient; multivariable linear regression analysis was performed applying a stepwise model including the following variables: gender, age, BMI, glucose, insulin, TG, HDL-cholesterol, ALT; variables entered the model if p < 0.05 and were removed if p > 0.1 An alpha level of p < 0.05 was considered significant for all statistical tests. Statistical analysis has been performed with the MedCalc Statistical Software version 14.12.0.

### Data Availability

All data generated or analyzed during this study are included in this published article.

## Results

### Patients’ characteristics

All the patients enrolled in the study presented with elevated blood pressure (hypertension): Table 1 shows a detailed list of the baseline characteristics of the study patients. About 36% of patients presented with three or more criteria used for MetS diagnosis according to the modified NCEP criteria, whereas the remaining part of the patients presented with one (hypertension) or two risk factors. As far as GGT is concerned, total and fractional GGT values were all within the normal reference values^12^. Table 2 shows the percentage distribution of MetS risk factors in the study population; the percentage of patients undergoing drug treatments relevant for MetS are also reported.

**Table 1:** Characteristics of the study patients.

Number of patients, n (M/W)	95 (60/35)
Age, years	56 (12)
BMI, kg/m^2^	27.4 (25.4–30.0)
Creatinine, mg/dL	0.96 (0.80–1.06)
Glucose, mg/dL	101 (94–109)
Insulin, μU/mL	8.6 (5.4–11.7)
Total cholesterol, mg/dL	187 (166–208)
HDL cholesterol, mg/dL	56 (46–67)
LDL cholesterol, mg/dL	117 (101–138)
Triglycerides, mg/dL	95 (69–126)
Total bilirubin, mg/dL	0.51 (0.40–0.67)
Direct bilirubin, mg/dL	0.20 (0.16–0.25)
AST, U/L	21(17–25)
ALT, U/L	21(15–29)
ALP, U/L	64 (54.0–77.5)
CRP, mg/dL	1.6 (0.77–3.30)
Leucocyte count (10^3^/μl)	6.4 (5.5–7.4)
Total GGT, U/L	17.8 (12.1–22.9)
b-GGT, U/L	1.9 (1.1–3.8)
m-GGT, U/L	0.4 (0.2–0.6)
s-GGT, U/L	4.3 (3.1–7.2)
f-GGT, U/L	9.6 (7.7–11.4)
b/s ratio	0.4 (0.3–0.6)

Data are presented as mean (SD) or as median (25^th^–75^th^ percentile). BMI, body mass index; TG, triglycerides; ALT, alanine aminotransferase; AST, aspartate aminotransferase; CRP, C-reactive protein.

**Table 2:** Data represent the number of patients (% of the total). BMI, body mass index; TG, triglycerides.

*Metabolic syndrome criteria*	
Hypertension	95 (100%)
Fasting glucose ≥ 100 mg/dL	52 (55%)
BMI M ≥ 29.5 kg/m^2^; F ≥ 27.2 kg/m^2^	33 (35%)
TG ≥ 150 mg/dL	14 (15%)
HDL M < 40 mg/dL; F < 50 mg/dl	13 (14%)
*Drugs:*	
Anti-hypertensives	85 (89%)
Calcium channel blockers	48 (50%)
Angiotensin receptor blockers	60 (63%)
Beta-blockers	20 (21%)
Angiotensin-converting enzyme (ACE) inhibitors	13 (14%)
Diuretics	35 (37%)
Lipid-lowering	
Statin	26 (27%)
Bezafibrate	4 (4.2%)
Glucose-lowering	
Metformin	8 (8.4%)
Vildagliptin	1 (1.1%)

### Fractional gamma-glutamyltransferase analysis and MetS risk factors

Among the four GGT fractions, b-GGT and f-GGT showed the highest positive Pearson’s correlation coefficient with serum levels of triglycerides, insulin, glucose, ALT and BMI (Table 3), and inverse correlation with HDL cholesterol. The highest correlations presented by s-GGT fraction were with BMI and ALT.

**Table 3:** Pearson’s correlation analysis.

	Variables					
	BMI	Glucose*	HDL	TG*	ALT*	Insulin*
b-GGT*	0.382^‡^	0.369^†^	−0.360^†^	0.472^‡^	0.353^†^	0.434^‡^
m-GGT*	0.266^§^	*0.199* ^*n.s*.^	−0.281^§^	0.218^#^	0.333^§^	0.224^#^
s-GGT*	0.312^§^	0.281^§^	−0.242^#^	0.203^#^	0.365^†^	0.282^§^
f-GGT*	0.452^‡^	0.438^‡^	−0.393^‡^	0.408^‡^	0.423^‡^	0.412^‡^
b-/s-GGT*	*0.192* ^*n.s*.^	*0.199* ^*n.s*.^	−0.251^#^	0.478^‡^	*0.079* ^*n.s*.^	0.315^§^

Data are Pearson correlation coefficients. BMI, body mass index; TG, triglycerides; ALT, alanine aminotransferase. *Statistical analysis was performed on ln-transformed data. Statistical significance level ^#^P < 0.05; ^§^P < 0.01; ^†^P < 0.001; ^‡^P < 0.0001; *n.s*. not significant.

Multiple regression analysis identified ALT as the common predictor of fractional GGT levels; triglycerides were the main predictor of b-GGT and f-GGT fraction and they were the sole predictor of the b-/s-GGT ratio. Moreover, insulin was independently associated only with b-GGT fraction (Table 4).

**Table 4:** Multivariable linear regression analysis.

	Variables								
	R^2^ adj	Gender M = 0 F = 1	Age	BMI	Glucose*	HDL	TG*	ALT*	Insulin*
b-GGT*	0.342	*n.s*.	*n.s*.	*n.s*.	*n.s*.	*n.s*.	0.419^‡^	0.295^§^	0.212^#^
m-GGT*	0.171	−0.234^#^	*n.s*.	*n.s*.	*n.s*.	*n.s*.	0.217^#^	0.297^§^	*n.s*.
s-GGT*	0.194	−0.232^#^	*n.s*.	0.221^#^	*n.s*.	*n.s*.	*n.s*.	0.265^#^	*n.s*.
f-GGT*	0.384	*n.s*.	*n.s*.	*n.s*.	0.239^#^	*n.s*.	0.324^§^	0.309^§^	*n.s*.
b-/s-GGT*	0.219	*n.s*.	*n.s*.	*n.s*.	*n.s*.	*n.s*.	0.477^‡^	*n.s*.	*n.s*.

Data are correlation coefficients adjusted for the effect of the other variables included in the model (partial correlation coefficient). BMI, body mass index; TG, triglycerides; ALT, alanine aminotranferase. *Statistical analysis was performed on ln-transformed data. Statistical significance level ^#^P < 0.05; ^§^P < 0.01; ^†^P < 0.001; ^‡^P < 0.0001; *n.s*. not significant.

In a first attempt to investigate the relationship between fractional GGT and MetS, patients were divided into two subgroups, *i.e*. those presenting with one or two MetS criteria (Control group, C) and those with three or more (MetS group). We found that b-GGT [C *vs*. MetS, median (25^th^-75^th^): 1.75 (0.95–2.85) U/L *vs*. 3.19 (1.50–6.59) U/L; p < 0.001], m-GGT [0.33 (0.19–0.60) U/L *vs*. 0.55 (0.26–0.81) U/L; p < 0.05], and f-GGT fraction activities [8.8 (7.0–10.6) U/L *vs*. 10.3 (9.1–13.6) U/L; p < 0.001] were significantly higher in subjects belonging to MetS group. Also the b-/s-GGT ratio was significantly higher [0.32 (0.26–0.54) U/L *vs*. 0.58 (0.41–0.78; p < 0.001] in MetS patients.

**Figure 1:**
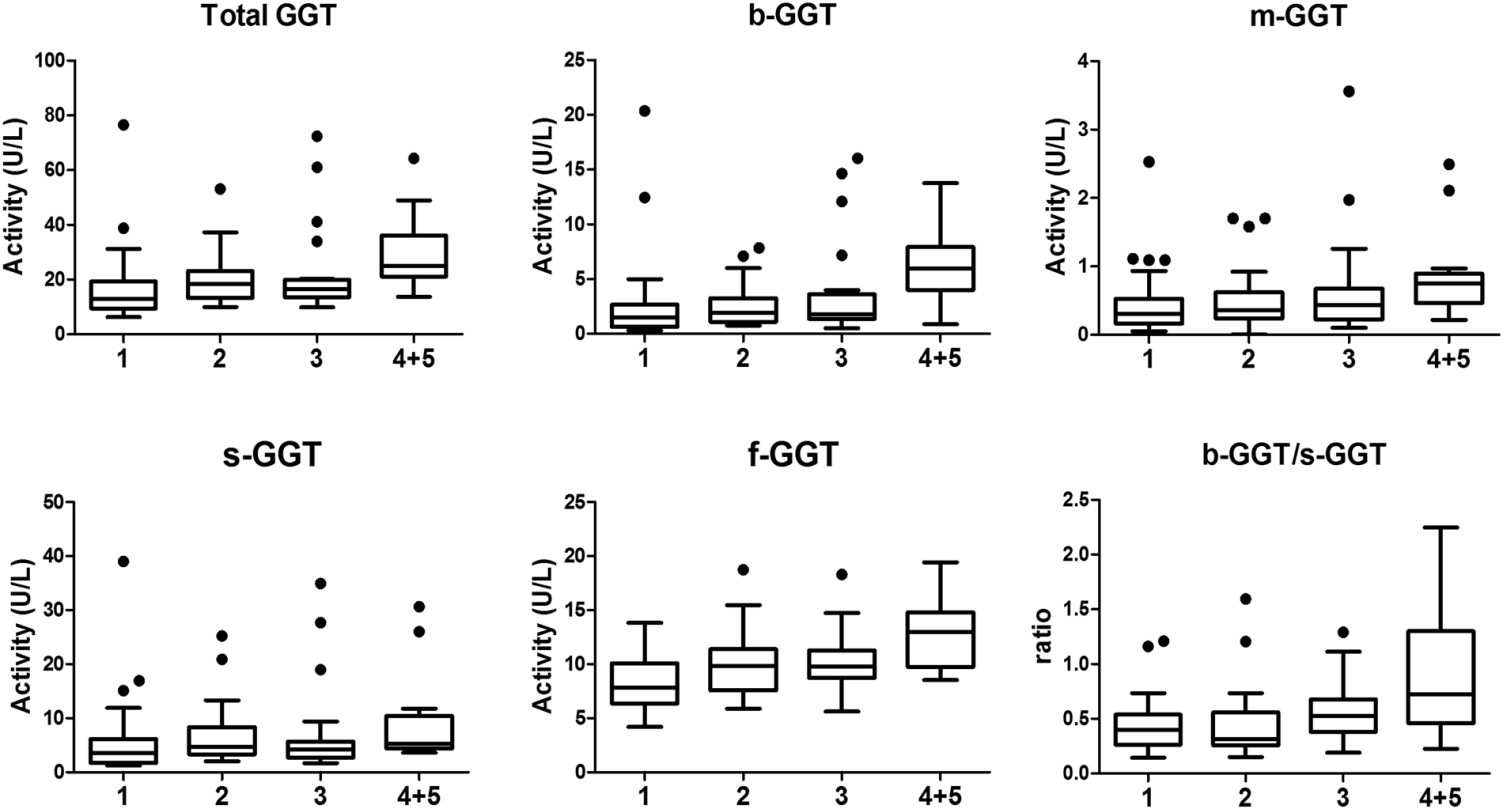
Distribution of fractional GGT activity in patients according to the number of the co-existing MetS criteria; patients with four or five criteria were grouped (“4 + 5”). The box represents the 25^th^ and 75^th^ percentiles and the line the median value. Whiskers correspond to the 25^th^ percentile minus 1.5 times IQR (interquartile range) and to the 75^th^ percentile plus 1.5 IQR.

On this background, patients were then divided into four subgroups, according to the number of co-existing MetS criteria. Due to the low number of patients, those with four or five criteria were grouped together (“4 + 5” subgroup). As shown in Fig. 1, the fractions b-GGT and f-GGT showed a significant increase along with the increase of the number of co-existing MetS criteria (P < 0.0001 for linear trend) and a similar trend was also observed for the b-/s-GGT *ratio* (P < 0.001). A further analysis revealed that the increase of values from subgroup “1” to subgroup “4 + 5” was more pronounced for b-GGT, as judged by the slope for the linear trend (b-GGT slope = 0.201 *vs* f-GGT slope = 0.068). The increase was more prominent between the subgroups “3” and “4 + 5”.

Since bezafibrate and metformin medications have been reported to lower serum total GGT activity^22–24^, we repeated all the analyses excluding the patients treated with these drugs (see Table 2). However, statistical analyses confirmed the results obtained in the whole group (data not shown).

### Fractional GGT analysis and liver steatosis

**Figure 2:**
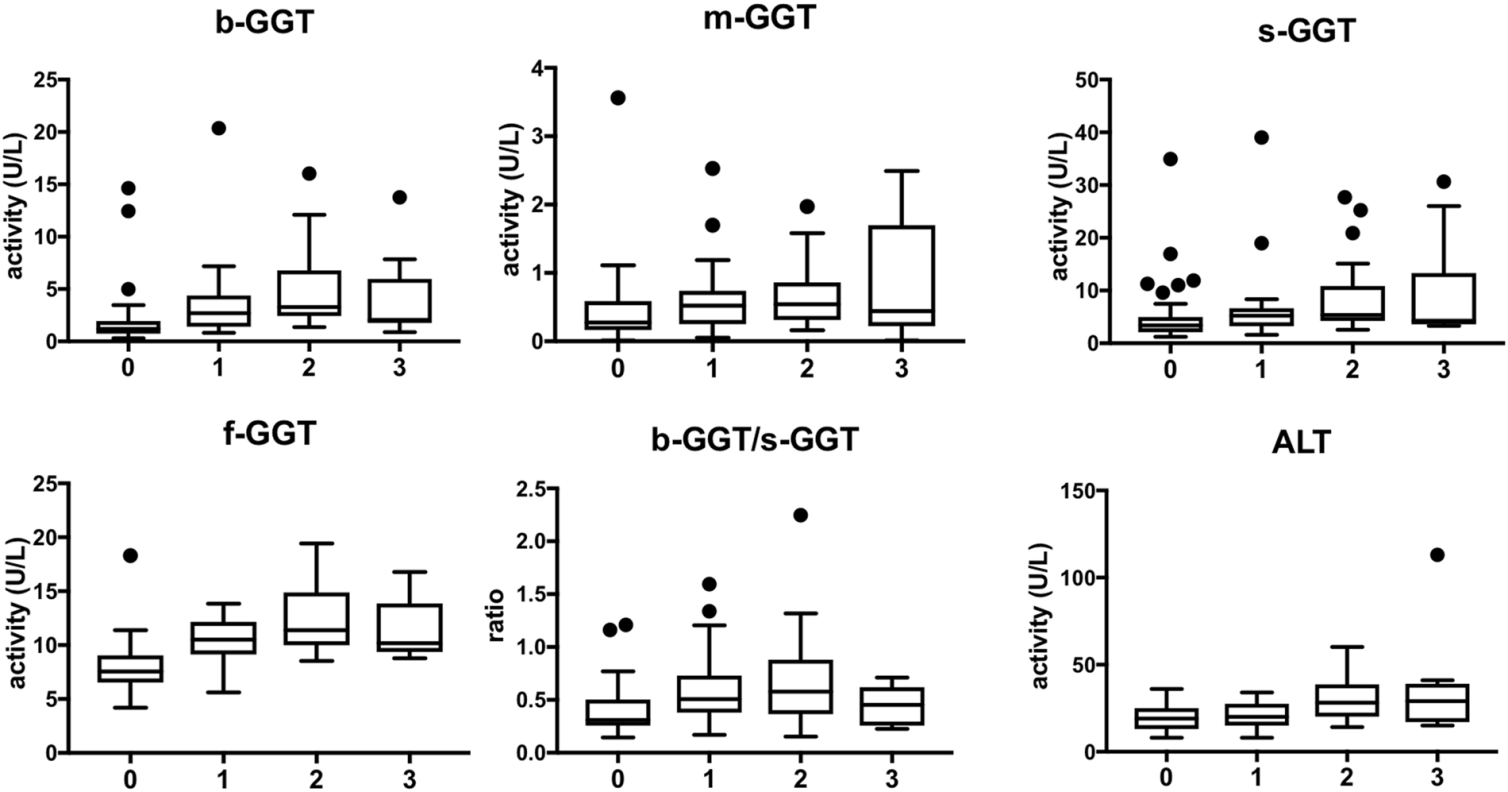
Distribution of fractional GGT activity, b-/s-GGT ratio and ALT according to the grade of liver steatosis. Tukey’s box and whiskers plot: the box extends from the 25^th^ to 75^th^ percentiles, the line in the middle is plotted at the median. Whiskers correspond to the 25^th^ percentile minus 1.5 times IQR (interquartile range) and to the 75^th^ percentile plus 1.5 IQR.

Patients were also grouped depending on the steatosis grade as evaluated by liver ultrasound (Fig. 2). The fractions b-GGT, s-GGT and f-GGT showed a progressive increase in patients with low (grade 1) or moderate liver steatosis (grade 2), in comparison with those without liver steatosis (grade 0), while no further increase was seen in patients with grade 3 steatosis (P < 0.0001, P < 0.01, P < 0.0001 for linear trend from grade 0 to grade 2). The b-/s-GGT ratio showed the same behavior (P < 0.001), while variation in m-GGT fraction levels were not statistically significant. The levels of ALT increased all over the subgroups according to the grade of steatosis (P < 0.001 for linear trend from grade 0 to grade 3). A further analysis revealed that the increase of values from grade 0 (g0) to grade 2 (g2) of steatosis was more pronounced for b-GGT median values (g0 = 1.19 U/L; g2 = 3.29 U/L; *ratio* g2/g0 = 2.75), as judged also by the slope for the linear trend (slope = 0.589). Indeed, lower increases were observed for s-GGT (g0 = 3.39 U/L; g2 = 5.37 U/L; *ratio* g2/g0 = 1.58; slope = 0.310), f-GGT (g0 = 7.54 U/L; g2 = 11.38 U/L; *ratio* g2/g0 = 1.50; slope = 0.231), b/s-GGT ratio (g0 = 0.31; g2 = 0.58; *ratio* g2/g0 = 1.87; slope = 0.278) and ALT (g0 = 19 U/L; g2 = 28 U/L; *ratio* g2/g0 = 1.47; slope = 0.173). Again, when patients treated with bezafibrate and metformin were excluded, statistical analyses confirmed the results obtained in the whole group (data not shown).

Finally, a two-way ANOVA was used to compare the effect of MetS presence and the grade of steatosis on fractional GGT values. The two-way ANOVA showed no significant interactions between the two factors but, interestingly, revealed only a statistically significant effect of the steatosis grade on b-GGT and f-GGT values (p < 0.01 and p < 0.001, respectively).

## Discussion

Among the risk factors associated to MetS, in Italy hypertension shows the highest prevalence^16,17^, and thus the identification of early, minimally invasive blood-based biomarkers for MetS diagnosis in hypertensive patients would be of great interest for their early risk stratification. In this perspective, the main findings of this study are that (i) hypertensive patients with three or more criteria for MetS display higher levels of b-GGT, m-GGT and f-GGT as compared to subjects with only one or two risk factors, but (ii) only b-GGT and f-GGT fractions significantly increase along with the number of MetS criteria presented. Accordingly, (iii) b-GGT and f-GGT fractions show the highest and significant correlations with all the others criteria associated to MetS, *i.e*. levels of triglycerides, glucose, HDL and BMI. These results are in good agreement with data from literature suggesting a positive correlation between total serum GGT activity and the onset of MetS (*e.g* ^4,7,25^). Interestingly our data show that b- and f-GGT are the main fractions that contribute to such correlation and that they are the most influenced fractions by liver steatosis, a condition known to be associated with MetS. Overall, our data confirm the higher diagnostic accuracy of fractional GGT analysis as compared to total GGT.

The plasma b-GGT fraction activity increases in NAFLD^13^, a condition frequently associated with insulin resistance^26^ and MetS^27^, and is positively associated with several cardiovascular risk factors including atherogenic dyslipidemia^15^. In this perspective our data also show a stronger increase of b-GGT median values - as compared to the other GGT fractions - along with the g0-g2 increase of steatosis grade.

The basis for such correlation might lie in the biogenesis of GGT fractions^28^. We found that the b-GGT corresponds to membrane microvesicles carrying the lipophilic GGT^29^ and that it is released *in vitro* by several cell types including the human hepatocyte HepG2 cell line^28^, and inflammatory cells upon activation^30,31^. Since all cell types found in the liver, *i.e*. hepatocytes, cholangiocytes, Kupffer, stellate and endothelial cells are able to release microvesicles^32,33^, and thus potentially b-GGT, further studies are required to establish the origin of b-GGT released during MetS and the contributions brought by the activated/damaged parenchymal liver cells and by the other cell types involved in liver inflammation.

The f-GGT is the most abundant fraction in healthy subjects^12^ and corresponds to a soluble and catalytically active form of GGT. We showed that f-GGT is obtained by proteolytic digestion of the other GGT fractions^28,29^. The f-GGT fraction is the simplest form of circulating GGT and its levels are likely to be influenced by the balance between production and catabolism of the other fractions^34^.

As regard the levels of s-GGT, we did not find any significant difference between patients with three or more criteria for MetS when compared to subjects with only one or two factors. We suggested that s-GGT may be constituted of bile-acid micelles carrying lipophilic GGT^29^ and we found that an increase in s-GGT levels is associated with hepatocellular damage, as observed in chronic viral hepatitis C and alcoholic-liver disease^13,35^. In confirmation of that, our results show that s-GGT is more significantly correlated with ALT levels rather than MetS factors. The increases of s-GGT and ALT between the second grade (g2) and the third grade (g3) of steatosis may be thus associated with the onset of a hepatocellular damage, and potentially to the onset of steatohepatitis. The ratio b-/s-GGT seems to mainly reflect the b-GGT behavior, thus suggesting the prevalence of metabolic alterations over the hepatocellular damage.

A s-GGT-like trend was observed also for the m-GGT fraction. Indeed, m-GGT levels – even higher in MetS group – did not show any significant increase along with the increase of MetS criteria and, accordingly, they showed a lower correlation with MetS factors when compared to b- and f-GGT. As for s-GGT, we have suggested that also m-GGT may be constituted of bile-acid micelles^29^: the similar pattern of biogenesis and their biochemical properties could thus help to explain the similar behavior of these two fractions. Further studies are however required to elucidate this specific point.

In conclusion our data showed that the investigation of fractional GGT could provide a novel, minimally invasive blood-based tool for a better identification of patients possibly presenting with the typical metabolic alterations of MetS, such as steatosis. Moreover, fractional GGT could be a useful tool for a better comprehension of MetS pathogenesis.
